# Supplementary material for: Mining Patents with Large Language Models Elucidates the Chemical Function Landscape
Source: ArXiv. 2023 Dec 18:arXiv:2309.08765v2. Preprint. [Version 2] (PMC10775343)
Supplement: 1 [file NIHPP2309.08765V2-supplement-1.pdf]

A Prompts

Patent summarization. The system prompt used was "You are an organic chemist summarizing chemical patents", and the user prompt was "Return a short set of three 1-3 word descriptors that best describe the chemical or pharmacological function(s) of the molecule described by the given patent title, abstract, and partial description (giving more weight to title & abstract). Be specific and concise, but not necessarily comprehensive (choose a small number of great descriptor). Follow the syntax '{descriptor_1} / {descriptor_2} / {etc}', writing 'NA' if nothing is provided. DO NOT BREAK THIS SYNTAX. The following is the patent:", followed by the patent title, abstract, and partial description.

Word embedding cluster summarization. Each cluster's labels were fed into GPT-3.5-turbo with the system prompt "You are a PhD pharmaceutical chemist" and the user prompt: "Given a set of molecular descriptors, return a single descriptor representing the centroid of the terms. Do not speculate. Only use the information provided. Be concise, not explaining answers. Example 1 Set of Descriptors: 11(beta)-hsd1, 11-hsd-2, 17β-hsd3 Example 1 Average Descriptor: hsd Example 2 Set of Descriptors: anti-retroviral, anti-retrovirus, anti-viral, anti-virus, antiretroviral, antiretrovirus, antiviral, antivirus Example 2 Average Descriptor: antiviral Set of Descriptors: __INSERT_DESCRIPTORS_HERE__ Average Descriptor:".

Graph label cluster summarization. Each cluster's labels were fed into GPT-4 with the system prompt "You are a PhD pharmaceutical chemist" and the user prompt: "Pretend you are a pharmaceutical chemist. I will provide you with several terms, and your job is to summarize the terms into appropriate categories. Be succinct, focusing on the broadest categories while still being representative. Don't show your work. Example terms: Antiviral HCV Kinase Cancer Polymerase Protease Example summarization: Antiviral & Cancer Terms: __INSERT_DESCRIPTORS_HERE__ Summarization:""".

B Supplemental data

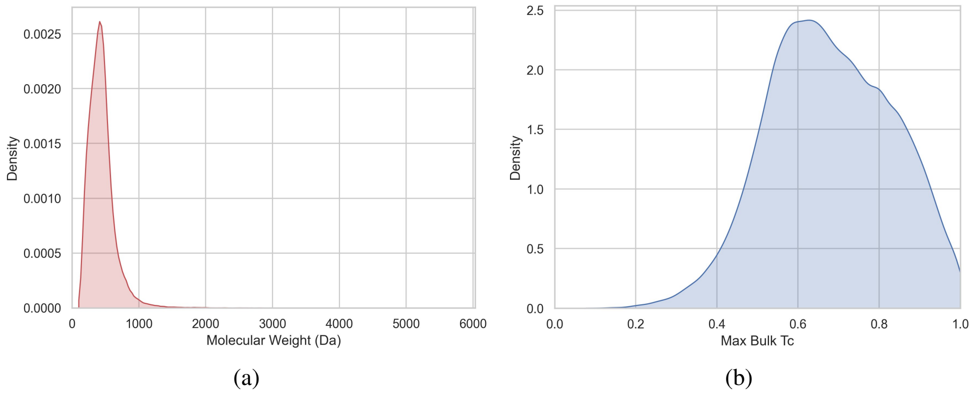

Figure S1: **Molecular weight and structural similarity distribution of the CheF dataset.** (a) Molecular weight of each molecule in the dataset. Minimum: 100.12 Da; Maximum: 5749.60 Da; Mean: 440.79 Da; Std: 203.96 Da. (b) Maximum bulk fingerprint Tanimoto coefficient (Tc) for each molecule in the dataset. Bulk Tc measures how similar a given molecule's structure is to all of the other molecules in the dataset. Max Bulk Tc returns the structural similarity of a molecule to the most structurally similar molecule in the dataset. High Max Bulk Tc indicates redundant structures, mid-low Max Bulk Tc indicates diverse structures. Minimum: 0.076; Maximum: 1.00; Mean: 0.68; Std: 0.15.

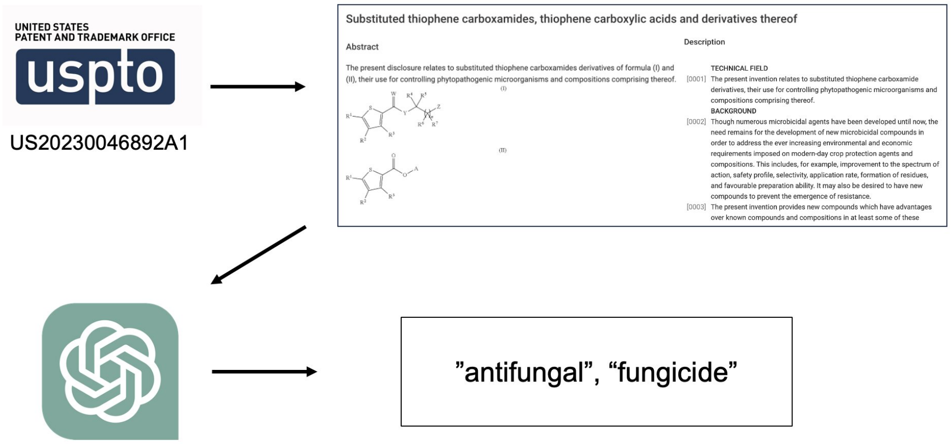

Figure S2: **Example of LLM-based chemical function extraction.** Patent IDs are used to retrieve the patent title, abstract, and description from Google Scholar. ChatGPT is then prompted to extract out the chemical function of the molecule being described by the patent.

Table S1: **ChatGPT patent summarization validation.** Manual validation was performed on 200 molecules randomly chosen from the CheF dataset. These 200 molecules had 596 valid associated patents, and 1,738 ChatGPT summarized labels. These labels were manually validated to determine the ratio of correct syntax, relevance to patent, and relevance to the Molecule of Interest (MOI).

Validation Task	Fraction Correct
Syntax	0.996
Label relevant to patent	0.998
Label refers to MOI, target of MOI, or downstream effects of MOI	0.779
Label refers to MOI, target of MOI, downstream effects of MOI, or molecules of which MOI is an intermediate	0.982

Table S2: **Validation of ChatGPT-aided label consolidation.** The first 500 of the 3,178 clusters of greater than one label (sorted in descending cluster size order) were evaluated for whether or not the clusters contained semantically common elements. The ChatGPT consolidated cluster labels were then analyzed for accuracy and representativeness. Common failure modes for clustering primarily included the grouping of grammatically similar, but not semantically similar labels (e.g., ahas-inhibiting, ikk-inhibiting). Failure modes for ChatGPT commonly included averaging the terms to the wrong shared common element (e.g., anti-fungal and anti-mycotic being consolidated to the label “anti”).

Validation Task	Fraction Correct
Cluster contains semantically common elements	0.992
ChatGPT cluster summarization accurate & representative	0.976

Table S3: **Comparison of Chemical-Text Datasets.** Comparison of CheF to existing chemical-text datasets ChEBI and ChemFOnt (Degtyarenko et al., 2007; Wishart et al., 2023) by current size (# molecules), maximum automated scaleup size (# molecules), text-type, whether or not structure and function are separate in the text, and the data source used for dataset construction. Both ChEBI and ChemFOnt were built from existing datasets with additional manual curation and annotation, limiting potential automated scaleup size. In contrast, the method used to build CheF scales readily, allowing for a potential dataset size of 32M molecules.

Dataset	Curr. Size	Scaleup Size	Text-Type	S/F Separate	Data Source
ChEBI	103K	103K+	Long text	No	DB Agg. / Manual
ChemFOnt	342K	1M+	Labels	Yes	DB Agg. / Manual
CheF (ours)	100K	32M+	Labels	Yes	LLM-Sum. Patents

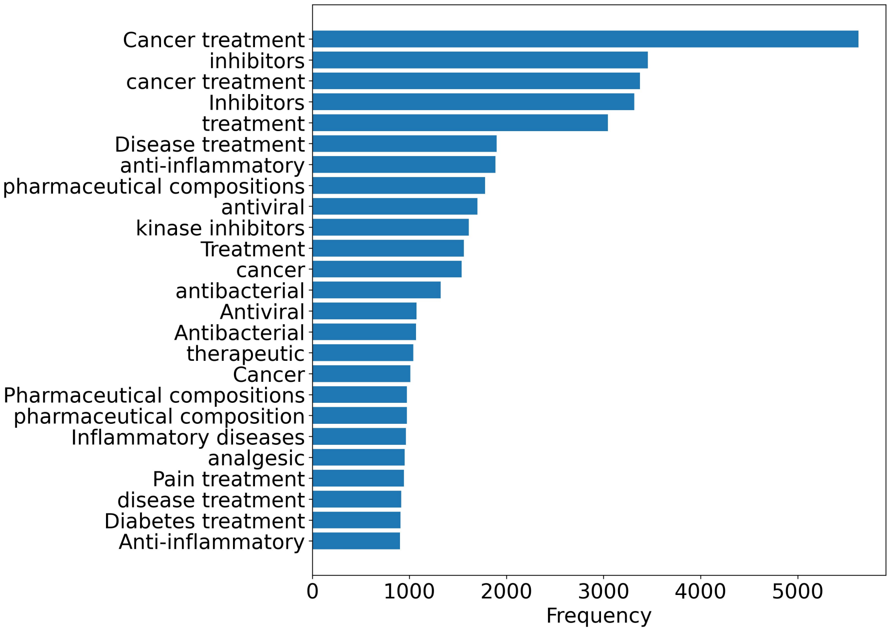

Figure S3: **Most frequent patent summarizations.** The most frequent patent summarizations do not immediately exhibit any dataset-independent biases. The bias towards broad treatment terms, such as cancer, antiviral, and analgesic, likely emerged because these are desirable target functions and are thus overrepresented in patents.

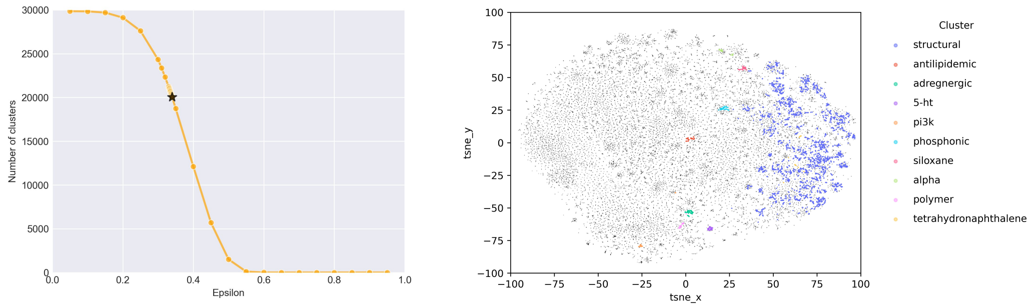

(a) (b)

Figure S4: **DBSCAN clustering on Ada-002 text embeddings reduces the number of labels.** (a) The optimal DBSCAN epsilon value was defined as the cutoff resulting in the smallest number of clusters without overtly false categories appearing (e.g., merging antiviral, antibacterial, & antifungal). The optimal epsilon was found to be 0.340 for the dataset considered herein (marked by black star), resulting in a consolidation from 29,854 labels to 20,030 clusters. The labels in each cluster were then consolidated with ChatGPT, creating a set of 20,030 labels. (b) t-SNE of the Ada-002 text embeddings, colored by the top 10 largest clusters. The largest cluster, found to be all IUPAC structural terms, was removed from the dataset to reduce excessive non-generalizable labels.

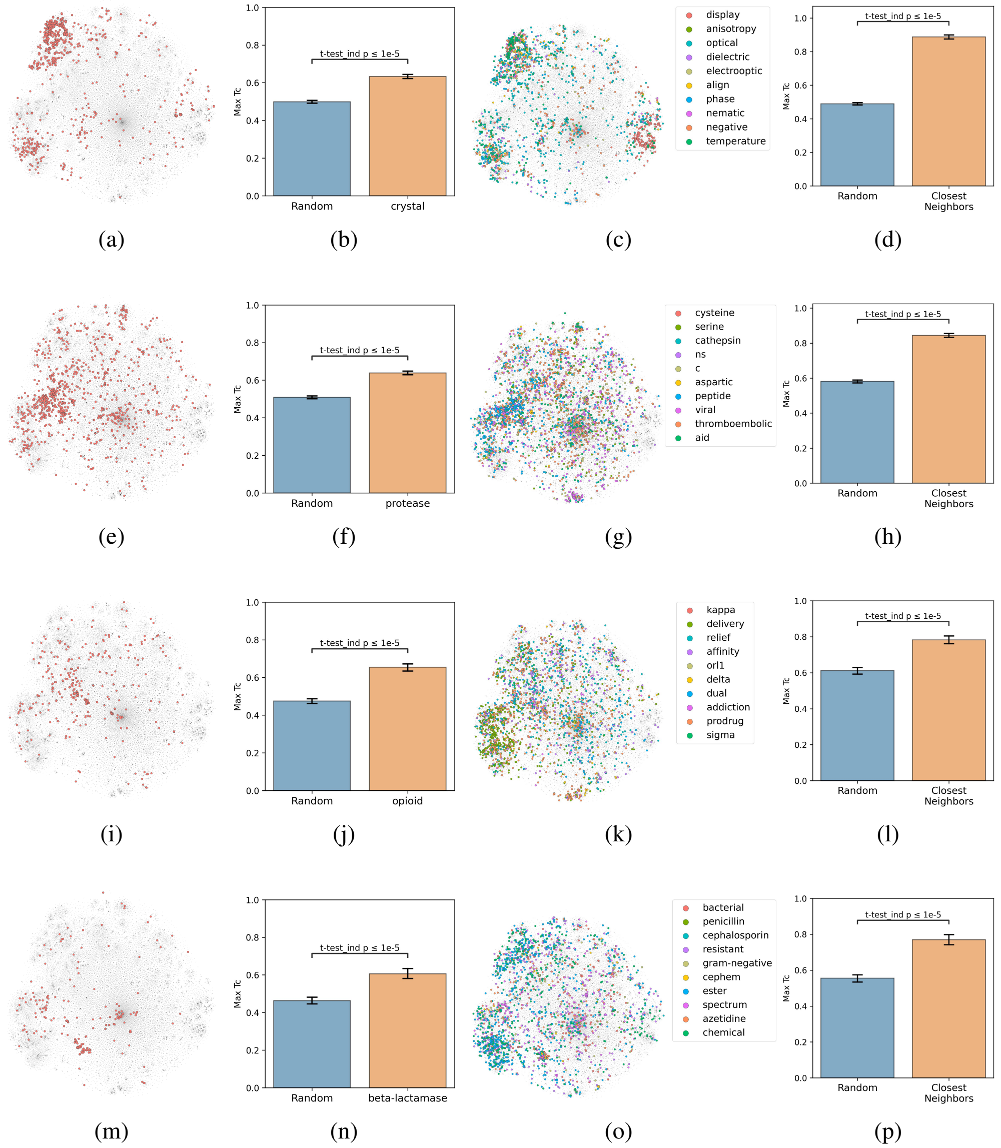

Figure S5: **Additional CheF labels and their clusters in structure space.** Molecules in the CheF dataset were projected based on molecular fingerprints and colored if the selected label was contained by the molecule's set of descriptors. To measure degree of clustering for a single label, the max fingerprint Tanimoto similarity from each molecule containing the selected label, to the other molecules containing that label, compared against the max fingerprint Tanimoto similarity for a random subset of molecules of the same size was obtained, whereas to measure the coincidence between the primary and co-occurring labels, the max fingerprint Tanimoto similarity from each molecule containing the primary label to each molecule containing any of the 10 nearest neighbor labels was compared against the max fingerprint Tanimoto similarity to a random subset of molecules of the same size. (a) Molecules containing label 'crystal'. (b) Degree of clustering for 'crystal'. (c) Molecules containing neighboring labels to 'crystal'. (d) Degree of coincidence between 'crystal' and its neighboring labels. (e) Molecules containing label 'protease'. (f) Degree of clustering for 'protease'. (g) Molecules containing neighboring labels to 'protease'. (h) Degree of coincidence between 'protease' and its neighboring labels. (i) Molecules containing label 'opioid'. (j) Degree of clustering for 'opioid'. (k) Molecules containing neighboring labels to 'opioid'. (l) Degree of coincidence between 'opioid' and its neighboring labels. (m) Molecules containing label 'beta-lactamase'. (n) Degree of clustering for 'beta-lactamase'. (o) Molecules containing neighboring labels to 'beta-lactamase'. (p) Degree of coincidence between 'beta-lactamase' and its neighboring labels.

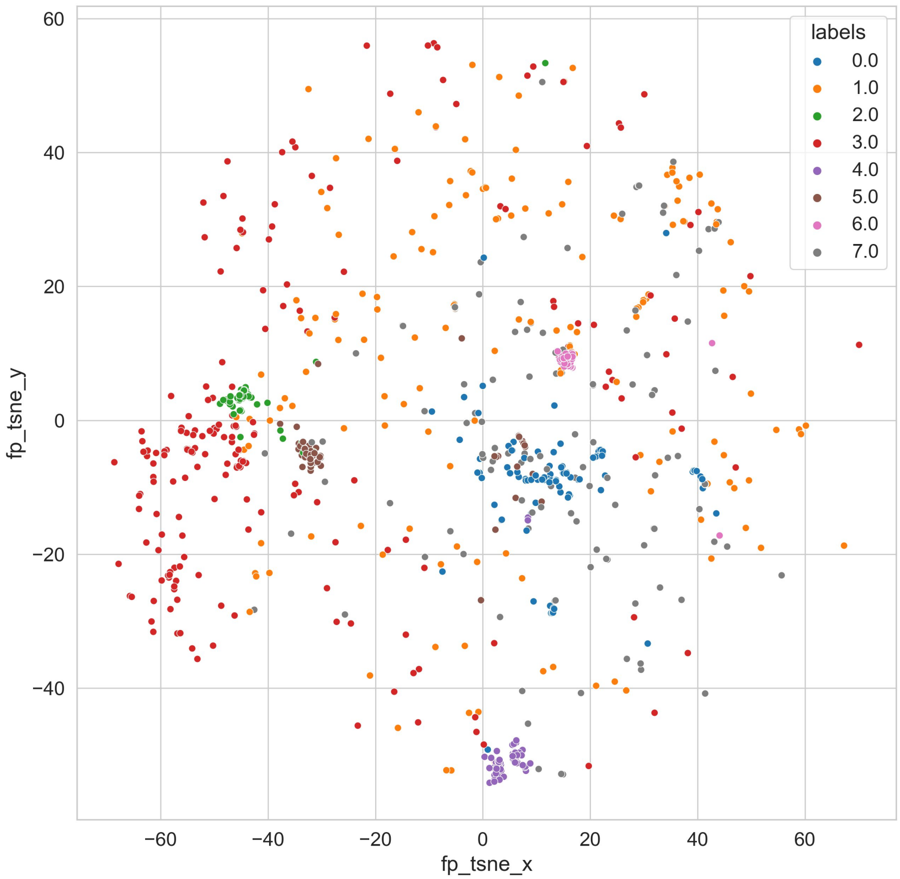

Figure S6: **K-means clustering on molecules containing ‘hcv’ elucidates Hepatitis C Virus (HCV) antiviral modalities.** The top 20 most frequently occurring labels were obtained for each of 8 clusters to determine their modalities (if applicable). Cluster 4 was the only cluster to contain ‘nucleoside’ (n=65) and ‘nucleotide’ (n=12) in the top 20 labels, indicating this cluster primarily contained HCV antiviral nucleoside derivatives likely inhibiting the NS5B polymerase. Cluster 2 contained ‘protease’ (n=85), ‘peptide’ (n=35), and ‘serine’ (n=15), indicating that this cluster primarily contained peptidomimetic protease inhibitors acting on the NS3 serine protease. Cluster 5 contained ‘protease’ (n=108), ‘macrocyclic’ (n=42), and serine (n=8), indicating that this cluster contained macrocyclic compounds acting likely as NS3 serine protease inhibitors. Cluster 6 contained no specific mechanistic terms, alluding to the possible mechanism of these molecules inhibiting the NS5A protein.

Table S4: **GPT-4 graph community summarizations.** All labels from the ten most abundant clusters were fed into GPT-4 for categorical summarization. These outputs were verified to be representative of the labels, and were further consolidated by the authors into concise categories.

**GPT4 Cluster summary**	**Label in graph**
Chemical Processes & Reactions, Materials & Substances, Photographic & Printing Processes, Cosmetic & Dermatological Applications, Industrial Manufacturing & Production, Sensory Properties	Material, Industrial, Synthesis, & Dermatology
Antiviral, Cancer, Cellular Processes, Enzymes, Immunology, Oncology, Protein Interactions, Therapy & Drug Development	Antiviral & Cancer
Pain Management, Hormonal Regulation, Gastrointestinal Conditions, Neurological Conditions, Reproductive Health, Obesity Management, Addiction Treatment, Sleep Disorders, Immune Response, Cardiovascular Conditions	Neurological, Hormonal, Gastrointestinal, & Reproductive Health
Chemical Compounds & Materials, Electronic & Optoelectronic Devices, Energy & Efficiency, Light & Emission Properties, Stability & Durability, Quantum & Thermodynamics	Electronic, Photochemical, & Stability
Neurodegenerative Diseases, Inflammatory & Autoimmune Diseases, Respiratory Diseases, Immune Response & Regulation, Enzymes & Mediators, Drug Development & Therapeutics	Neurodegenerative, Autoimmune, Inflammation, & Respiratory
Antibacterial, Antifungal, Antiparasitic, Antimalarial, Antimicrobial, Antiprotozoal, Antitubercular, Insecticide, Herbicide, Fungicide, Pesticide, Acaricide, Nematicidal, Agricultural & Health Protection	Anti-Organism & Agricultural
Drug Development & Delivery, Diagnostic & Monitoring, Gene & Protein Regulation, Epigenetics & Transcription, Immunology & Vaccines	Pharmaceutical Research, Genetic Regulation, Immunology
Neurological & Psychiatric Disorders, Cognitive & Memory Function, Neuropharmacology & Neurotransmission, Mood & Mental Health, Urologic & Sexual Health	Neurological & Urologic
Lipid Metabolism & Cardiovascular Health, Diabetes Management, Organ Health & Protection	Cardiovascular & Lipid Metabolism
Cardiovascular & Renal Disorders, Ion Channels & Transporters, Anesthetics & Muscle Relaxants, Neurological Disorders & Eye Conditions	Cardiovascular, Renal, & Ion Channel

Table S5: **Arbitrary 20 CheF labels from each summarized co-occurrence neighborhood.** Modularity-based community detection was performed on the CheF co-occurrence graph to obtain 19 distinct communities. The communities appeared to broadly coincide with the semantic meaning of the contained labels, and the largest 10 communities were summarized to a common label. Shown are a random 20 labels from the first five summarized communities.

**Material, Industrial, Synthesis, & Dermatology**	**Antiviral & Cancer**	**Neurological, Hormonal, Gastrointestinal, & Reproductive Health**	**Electronic, Photochemical, & Stability**	**Neuro-degenerative, Autoimmune, Inflammation, & Respiratory**
absorb	aid	analgesic	carbazole	activate
acid	antiviral	condition	compound	adhesion
binder	c	ligand	expand	alzheimer
care	cancer	modulate	life	amyloid
cosmetic	cell	modulator	light	anti-inflammatory
destabilize	g12	p2x7	material	autoimmune
form	hbv	pain	activated	cox
functional	hcv	prophylaxis	amine	disease
ionic	hepatitis	prostate	anisotropy	elastase
method	hiv	receptor	aromatic	il-17
modification	inhibit	relief	blue	inflammation
optical	inhibition	selective	capability	inflammatory
photochromic	inhibitor	tgr5	characteristic	interferon
plastic	integrase	tract	charge	lung
polymer	kinase	treatment	condensed	neuro-degenerative
preserve	kras	various	crystal	neuro-inflammation
production	mapk	5-ht	cyclic	sting
protective	nucleoside	7	device	airway
sensitivity	phosphatidyl-inositol	addiction	dielectric	allergic
skin	phosphorylation	adrenergic	diode	allergy

Table S6: **Arbitrary 20 CheF labels from each summarized co-occurrence neighborhood.** Modularity-based community detection was performed on the CheF co-occurrence graph to obtain 19 distinct communities. The communities appeared to broadly coincide with the semantic meaning of the contained labels, and the largest 10 communities were summarized to a common label. Shown are a random 20 labels from the second five summarized communities.

**Anti-Organism & Agricultural**	**Pharmaceutical Research, Genetic Regulation, Immunology**	**Neurological & Urologic**	**Cardiovascular & Lipid Metabolism**	**Cardiovascular, Renal, & Ion Channel**
amide	assay	anticonvulsant	carbonic	cardiovascular
control	bind	cerebral	ischemia	channel
derivative	bromodomain	disorder	level	ion
infection	diagnostic	function	liver	stroke
protection	drug	mitochondrial	prevention	ace
acaricide	potential	neural	reducer	anesthetic
acetic	psma	neuroprotective	reducing	angina
animal	regulator	pde	reduction	angiotensin
anti	sirtuin	schizophrenia	regulate	anti-hypertensive
anti-malarial	targeting	sedative	releasing	blocker
anti-microbial	6	system	retinoid	calcium
antiparasitic	alter	urologic	vap	cardiac
aryl	analog	4	vascular	cardiotonic
azetidin	atp	5	a	circulation
azetidine	atrophy	anti-psychotic	aldose	c-transport
bacterial	bioavailability	antidepressant	alleviate	contraction
bactericide	biological	antitussive	antilipidemic	diuretic
beta-lactamase	biomarker	anxiolytic	blood	failure
bicyclic	combinatorial	brain	cholesterol	heart
bridge	cytotoxic	central	cholesterolemia	hypertensive

Table S7: **Fingerprint models benchmarked on CheF.** To assess a baseline benchmark on the CheF dataset of ~100K molecules, several molecular fingerprint-based models were trained on 90% of the training data and evaluated on the 10% test set holdout. Macro average ROC-AUC and PR-AUC was calculated across all 1,543 labels. Logistic regression (LR), random forest classifier (RFC), and a 2-layer feedforward neural network (FFN) were trained. Parameters for LR and RFC were chosen to be common default values, whereas the FFN layer number and size were chosen through a 5-fold cross validation.

**Model**	**ROC-AUC**	**PR-AUC**
FP + LR	**0.84**	**0.20**
FP + RFC	0.80	0.13
FP + FFN	0.81	0.12

Name	P(hcv)	P(hepatitis)	P(antiviral)	P(ns)	P(protease)	P(polymerase)	P(ace)	P(btk)
DACLATASVIR DIHYDROCHLORIDE	0.95	0.42	0.95	0.84	0.02	0.00	0.00	0.01
DACLATASVIR	0.95	0.42	0.95	0.84	0.02	0.00	0.00	0.01
GRAZOPREVIR	0.94	0.41	0.81	0.59	0.73	0.00	0.00	0.01
BOCEPREVIR	0.91	0.73	0.42	0.03	0.88	0.00	0.00	0.00
PARITAPREVIR	0.83	0.12	0.75	0.67	0.73	0.00	0.00	0.00
SIMEPREVIR	0.83	0.06	0.35	0.09	0.07	0.01	0.00	0.00
SIMEPREVIR SODIUM	0.83	0.06	0.35	0.09	0.07	0.01	0.00	0.00
VOXILAPREVIR	0.75	0.29	0.65	0.24	0.34	0.00	0.00	0.00
SOFOSBUVIR	0.70	0.16	0.86	0.03	0.00	0.23	0.00	0.00
LEDIPASVIR	0.67	0.53	0.86	0.15	0.01	0.00	0.00	0.00
ELBASVIR	0.61	0.58	0.83	0.15	0.01	0.01	0.00	0.00
GLECAPREVIR	0.56	0.15	0.63	0.26	0.23	0.00	0.00	0.01
VELPATASVIR	0.54	0.68	0.81	0.16	0.02	0.00	0.00	0.01
OMBITASVIR	0.41	0.25	0.41	0.02	0.12	0.00	0.00	0.00
NELARABINE	0.33	0.05	0.45	0.00	0.00	0.01	0.00	0.00
PIBRENTASVIR	0.20	0.32	0.35	0.02	0.01	0.00	0.00	0.01
BAZEDOXIFENE	0.10	0.07	0.07	0.01	0.01	0.01	0.00	0.00
BAZEDOXIFENE ACETATE	0.10	0.07	0.07	0.01	0.01	0.01	0.00	0.00
TEGAFUR	0.09	0.02	0.43	0.00	0.01	0.01	0.00	0.00
MICAFUNGIN	0.08	0.04	0.08	0.03	0.19	0.01	0.00	0.00
MICAFUNGIN SODIUM	0.08	0.04	0.08	0.03	0.19	0.01	0.00	0.00
ACYCLOVIR SODIUM	0.08	0.02	0.74	0.00	0.00	0.01	0.00	0.00
RUCAPARIB CAMSYLATE	0.08	0.09	0.10	0.03	0.00	0.01	0.00	0.01
PERINDOPRIL ERBUMINE	0.08	0.08	0.11	0.02	0.06	0.00	0.20	0.00
DARIDOREXANT HYDROCHLORIDE	0.08	0.03	0.07	0.03	0.00	0.00	0.00	0.01
DARIDOREXANT	0.08	0.03	0.07	0.03	0.00	0.00	0.00	0.01
ACALABRUTINIB	0.07	0.06	0.23	0.09	0.03	0.00	0.00	0.78
PERINDOPRIL ARGININE	0.07	0.07	0.11	0.02	0.06	0.00	0.15	0.00
CYTARABINE	0.07	0.01	0.32	0.00	0.00	0.02	0.00	0.00
PERINDOPRIL	0.07	0.07	0.11	0.02	0.07	0.00	0.20	0.00
IDOXURIDINE	0.07	0.01	0.43	0.00	0.01	0.03	0.00	0.00
MARIBAVIR	0.07	0.04	0.20	0.00	0.00	0.01	0.00	0.00
ACALABRUTINIB MALEATE	0.07	0.05	0.24	0.09	0.02	0.00	0.00	0.78
ADENOSINE PHOSPHATE	0.07	0.03	0.17	0.00	0.00	0.02	0.00	0.00
GANCICLOVIR SODIUM	0.07	0.01	0.70	0.00	0.00	0.01	0.00	0.00
REMDESIVIR	0.06	0.07	0.82	0.02	0.01	0.04	0.00	0.00
FLUDARABINE PHOSPHATE	0.06	0.03	0.17	0.00	0.00	0.01	0.00	0.00
GEMCITABINE	0.06	0.02	0.66	0.00	0.00	0.02	0.00	0.00
GEMCITABINE HYDROCHLORIDE	0.06	0.02	0.66	0.00	0.00	0.02	0.00	0.00
URIDINE TRIACETATE	0.06	0.01	0.25	0.00	0.00	0.02	0.00	0.00
RUCAPARIB	0.06	0.10	0.15	0.02	0.00	0.01	0.00	0.01
PENCICLOVIR	0.06	0.03	0.50	0.00	0.00	0.02	0.00	0.00
AMDINOCILLIN PIVOXIL	0.05	0.01	0.04	0.01	0.02	0.01	0.00	0.00
CAPECITABINE	0.05	0.01	0.25	0.00	0.00	0.01	0.00	0.00
FLOXURIDINE	0.05	0.01	0.41	0.00	0.00	0.02	0.00	0.00
BRIVARACETAM	0.05	0.01	0.07	0.01	0.05	0.00	0.00	0.00
DITHIAZANINE	0.05	0.02	0.07	0.00	0.01	0.00	0.00	0.00
BRINZOLAMIDE	0.05	0.08	0.15	0.02	0.02	0.01	0.00	0.00
ENASIDENIB	0.04	0.04	0.05	0.00	0.00	0.00	0.00	0.00
ANIDULAFUNGIN	0.04	0.05	0.12	0.01	0.22	0.00	0.00	0.00

Figure S7: **Top 50 FDA-approved drugs predicted to contain the label 'hcv'.** The Stage-4 approved drugs list from OpenTargets was passed through the CheF label prediction model. Results were sorted by 'hcv' probability. Relevant and high abundance labels displayed for clarity. Green cells represent approved-use labels from on the OpenTargets page, and red cells represent no approved usage relevant to the given term.
